# Therapeutic Targeting of β-Catenin in Cancer

**DOI:** 10.1158/1535-7163.MCT-26-0062

**Published:** 2026-07-15

**Authors:** Paola Martinelli, Peter Repiscak, Norbert Kraut

**Affiliations:** Boehringer Ingelheim RCV GmbH & Co KG, Vienna, Austria.

## Abstract

Mutations in *CTNNB1* (encoding β-catenin), together with recurrent genetic alterations affecting the WNT signaling pathway, define one of the most prevalent oncogenic axes, collectively occurring in approximately 10% of human cancers. Thus, β-catenin is a prime target for precision oncology. Over the three decades following the pathway’s discovery, substantial progress has been made in elucidating how aberrant WNT/β-catenin signaling can drive cancer initiation, progression, and maintenance. Attempts to therapeutically address this pathway have, until recently, been unsuccessful. Approaches to block the pathway upstream of relevant mutations were ineffective, whereas progress in directly targeting β-catenin, either by blocking its interaction with key complex partners, most notably T-cell factor proteins, or by inducing β-catenin degradation, has long remained an unreachable goal for conventional drug discovery approaches. In this review, we describe opportunities and challenges in the development of therapies that directly aim to target β-catenin. We highlight recent progress based on novel approaches, suggesting that cracking this previously considered “undruggable” central oncogenic driver is becoming a reality. Effective targeting of β-catenin has the potential to address extensive unmet patient needs in hard-to-treat cancer types such as colorectal cancer, hepatocellular carcinoma, and other cancers driven by WNT/β-catenin pathway alterations.

## Introduction

The canonical WNT/β-catenin signaling pathway is a highly conserved developmental signal transduction cascade governing cell fate determination, tissue homeostasis, and stem cell maintenance. In the absence of WNT ligands, cytoplasmic β-catenin levels are maintained at low levels through the balance of synthesis and fast proteasomal degradation by the destruction complex. This complex, composed of the tumor suppressors adenomatosis polyposis coli (APC) and AXIN1/AXIN2, as well as the kinases casein kinase 1 (CK1) and glycogen synthase kinase 3β (GSK3β), phosphorylates β-catenin and promotes its ubiquitination and subsequent degradation. The engagement of WNT ligands with Frizzled (FZD) receptors and low-density lipoprotein receptor–related proteins 5 and 6 (LRP5/6) coreceptors inhibits the destruction complex’s activity, allowing β-catenin to accumulate, translocate to the nucleus, and associate with T-cell factor (TCF)/lymphoid enhancer factor transcription factors to drive context-dependent gene expression programs. The magnitude of WNT signaling is further regulated at the cell surface by several key modulators. Porcupine (PORCN) is required for the secretion of WNT ligands, and R-spondins (RSPO1–4) potentiate WNT signaling by neutralizing the transmembrane E3 ubiquitin ligases RING finger protein 43 (RNF43) and zinc and RING finger 3 (ZNRF3), which normally promote the internalization and degradation of FZD receptors, thereby limiting pathway activation. Thus, stabilized β-catenin is the effector of canonical WNT signaling, orchestrating pathway-induced gene transcription (Fig. 1). A more detailed description of the pathway can be found in several reviews (1–4). Here, we will focus on the role of the WNT/β-catenin pathway in cancer.

**Figure 1.**
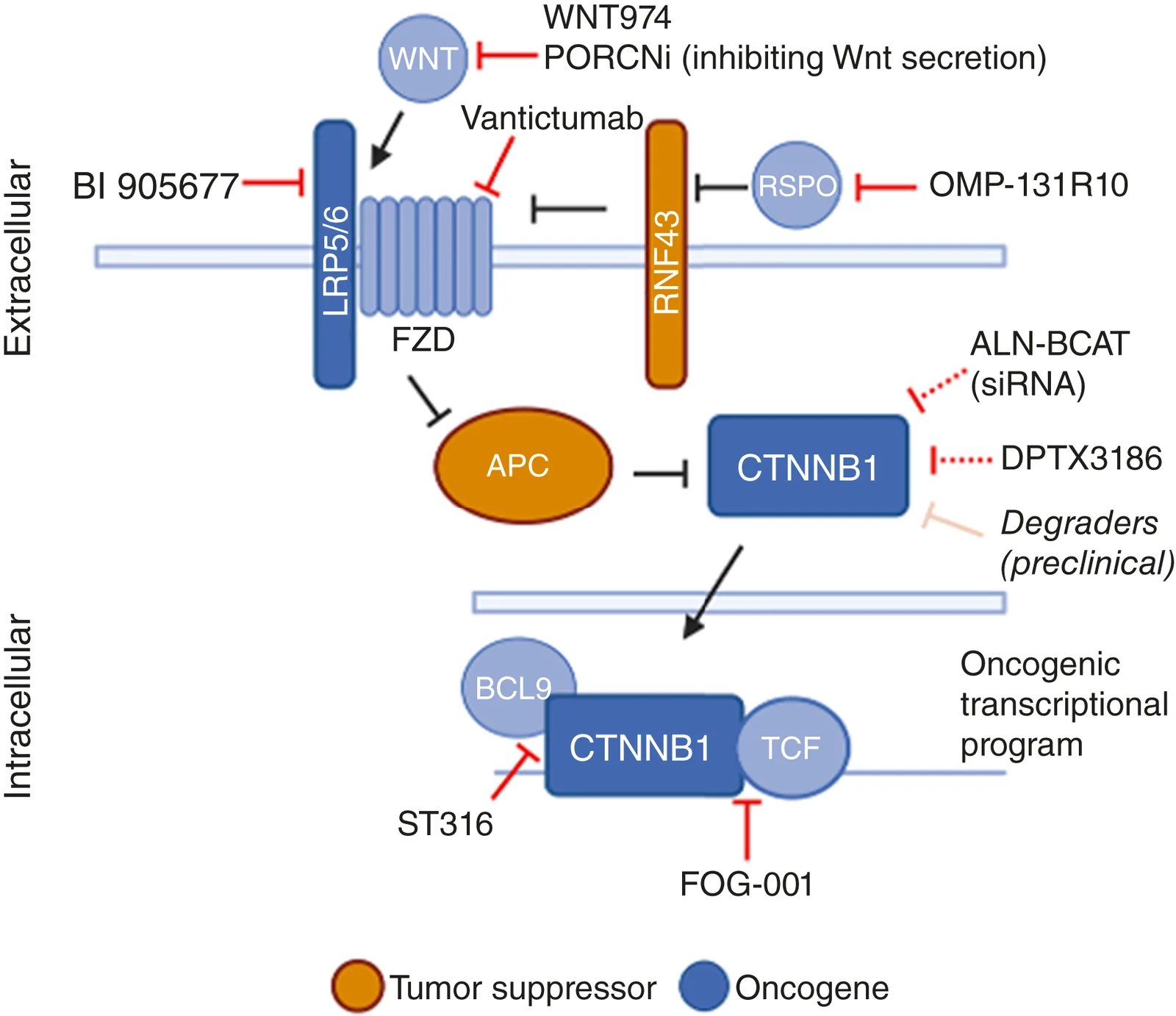
Targeting the WNT/β-catenin pathway in the clinic. A simplified scheme of the canonical WNT/β-catenin signaling cascade, indicating some of the inhibitors that have been clinically tested so far. ALN-BCAT and DPTX3186 are represented with a dotted line to indicate that they do not directly target the β-catenin protein but rather its mRNA (ALN-BCAT) or an undisclosed protein that regulates nuclear condensates (DPTX3186).

In healthy tissues, tight regulation of the WNT/β-catenin pathway is critical for maintaining tissue homeostasis. Unsurprisingly, genetic alterations in the pathway that result in ligand-independent stabilization of β-catenin are linked to several growth-related diseases, including cancer. Although loss-of-function truncating mutations in *APC* primarily occur in colorectal cancer (5, 6), activating mutations in *CTNNB1*, typically affecting the N-terminal phospho-degron required for β-catenin turnover, are prevalent in hepatocellular carcinoma (HCC; refs. 7, 8), desmoid tumors (9), and subsets of other solid malignancies. Less frequently, inactivating alterations in *AXIN1* or *AXIN2* contribute to aberrant pathway activation (Fig. 2A). Collectively, these lesions converge on persistent β-catenin stabilization and transcriptional output.

**Figure 2.**
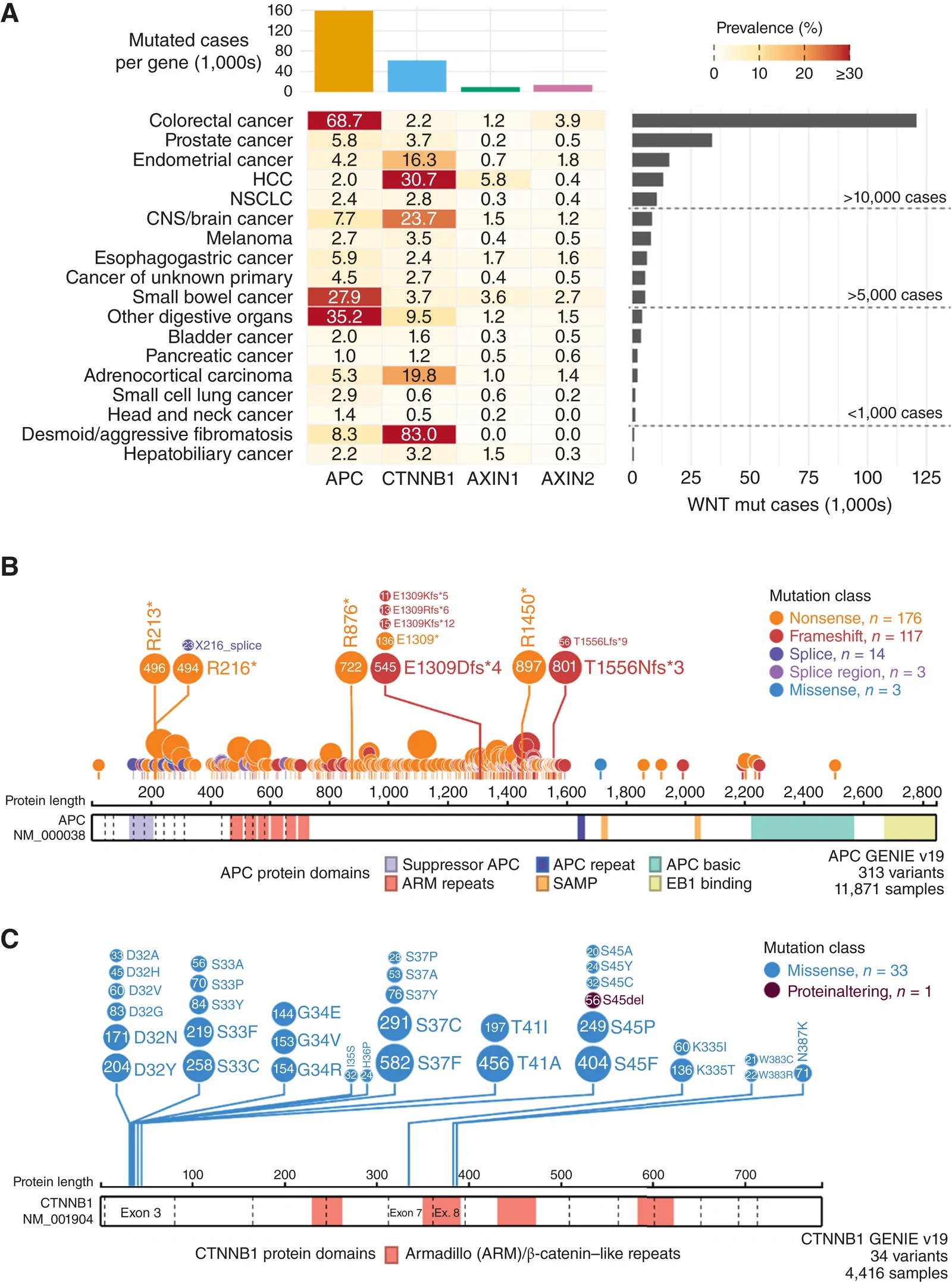
WNT pathway mutation landscape across cancers and gene-level mutation profiles. **A,** Distribution of relative prevalences (%) of oncogenic [as defined by OncoKB (10, 11) or HotSpot (12)] *APC*, *CTNNB1*, *AXIN1* and *AXIN2* mutations across cancer types derived from the AACR GENIE v19.0-public (13) database (*n* = 181,948 *CTNNB1*; 180,296 *APC*; 133,159 *AXIN1*; 143,952 *AXIN2* profiled patient samples), with corresponding estimated patient numbers. Patient numbers are based on estimated US incident cases for 2026 [Cancer Facts & Figures 2026 report (14)], incorporating histology- and grouping-based adjustments (e.g., HCC ≈ 80%, hepatobiliary ≈ 20% of liver; NSCLC ≈ 77%, small cell lung cancer ≈ 13% of lung; esophagogastric cancer from combined esophagus + stomach). Prevalences for selected digestive cancers were grouped as “Other digestive organ cancers,” and central nervous system (CNS)–related entities were grouped as “CNS/brain cancer.” For desmoid tumors, an estimate of ∼900 new cases annually in the United States was used (15). Cancer types shown meet ≥1,000 annual cases or ≥5% combined prevalence across the four WNT pathway genes. **B** and **C,** Mutation profiles of *APC* (**B**) and *CTNNB1* (**C**) from AACR GENIE v19.0-public visualized with ProteinPaint (16). Discs represent individual mutations, scaled by the number of affected patients and colored by mutation class. Mutations are positioned along the protein by amino acid coordinate, with stacked discs indicating recurrent hits at the same residue. Only recurrent mutations (≥10 cases) are shown; amino acid changes and counts are annotated. Dashed vertical lines inside the protein denote exon boundaries, and annotated domains are indicated in the legend. An asterisk (*) indicates a translation termination (stop) codon, following the Human Genome Variation Society (HGVS) nomenclature for sequence variant annotation.

Beyond its cancer cell–intrinsic role in driving proliferation, survival, and stemness, the WNT/β-catenin pathway has emerged as a key regulator of antitumor immunity. Several lines of evidence originating from the analysis of cell lines (17), patient-derived samples (18), as well as mouse models of melanoma and liver cancer (18, 19), revealed that tumor cell–intrinsic β-catenin activation is linked to immune exclusion through impaired dendritic cell recruitment, reduced T-cell priming, and resistance to immune checkpoint blockade. Of note, the immunosuppressive role of the activated WNT/β-catenin pathway might not be a universal feature in cancer, as exemplified by the autocrine signaling suppression driven by the WNT inhibitor Dickkopf-related protein 1 (DKK1) in quiescent stem-like tumor cells with latent metastatic potential, which downregulated natural killer cell ligands, thus evading immune surveillance (20).

The first TCF gene was cloned in T cells [*TCF7*, coding for the TCF1 protein (21)] and was shown to function in part through the WNT/β-catenin signaling pathway (22). Consistently, on top of the tumor-intrinsic role in immune evasion, stabilized β-catenin also has immune cell–intrinsic functions, such as improving the survival of regulatory T cells (23), driving dendritic cells into a more tolerogenic regulatory state (24), and impairing the differentiation of CD8^+^ effector T cells (25). Therefore, a therapeutic approach targeting β-catenin may directly affect immune cells.

These dual oncogenic and immunomodulatory functions position β-catenin as a central driver of both tumor progression and immune evasion. Consequently, therapeutic strategies that effectively target β-catenin signaling hold the promise not only of direct antitumor activity but also of restoring antitumor immunity in WNT/β-catenin–driven cancers.

## The WNT/β-Catenin Pathway is Commonly Altered in Cancer

Mutations in *APC*, *CTNNB1*, *AXIN1*, and *AXIN2* are largely mutually exclusive across cancers, supporting their convergence on the aberrant activation of the WNT/β-catenin signaling pathway. A detailed analysis of the molecular relevance of the various mutations in the WNT/β-catenin pathway can be found in a review by Bugter and colleagues (26).

In brief, *APC* is most frequently affected by loss-of-function truncating mutations (nonsense or frameshift), typically clustered within the mutation cluster region located between codons 1282 and 1581 (Fig. 2B; ref. 27). As APC acts as a scaffold for the β-catenin destruction complex, these alterations impair proper assembly of the complex, thus preventing phosphorylation and subsequent degradation of β-catenin. In contrast, *CTNNB1* harbors hotspot missense mutations, predominantly affecting serine/threonine residues in exon 3 that are required for GSK3β- and CK1-mediated phosphorylation (Fig. 2C; ref. 28). *AXIN1* and *AXIN2*, which encode scaffold proteins essential for the destruction complex assembly, are less frequently mutated but exhibit loss-of-function alterations, including truncations and destabilizing missense mutations (further details on *AXIN1* and *AXIN2* mutations are not shown). Based on the analysis of prevalence from the American Association for Cancer Research (AACR) project GENIE [release 19.0-public (13)] data and estimated new yearly cancer cases in the United States as extracted from the Cancer Facts & Figures 2026 report published by the American Cancer Society (14), we estimate approximately 244,000 new annual diagnoses/patients with alterations in one of the four genes (*APC*, *CTNNB1*, *AXIN1*, *AXIN2*; see Fig. 2A) across all nonhematologic cancers.

Several lines of evidence suggest that the WNT/β-catenin pathway can also be activated in a mutation-independent manner in various cancer types, for example, through overexpression of WNT ligands, both by cancer cells and cells in the tumor microenvironment, thereby supporting metastatic progression (29–32). Therefore, the number of patients who could benefit from a WNT/β-catenin–targeted therapy is potentially even larger than detailed above. However, the identification of patients with nongenetic activation of the pathway will require a more complex diagnostic test.

## β-Catenin is a Central Cancer Dependency

The role of the WNT/β-catenin pathway was experimentally confirmed through the generation of several genetically engineered mouse models (GEMM) for colorectal cancer and HCC. In mice, conditional expression of a stabilized β-catenin protein lacking exon 3—in which the phosphodegron region is located—in the intestinal epithelium causes adenomatous intestinal polyps and nascent microadenomas (33). Similarly, loss of *Apc* in Lgr5-positive stem cells of the intestine through conditional deletion (34) or short hairpin RNA (shRNA)–mediated knockdown (35) is sufficient to initiate the formation of adenomas in the small intestine or both in the small intestine and the colon, respectively. Additional alterations such as *Kras*^G12D/V^ or *p53* loss (common comutations in patients) can drive adenocarcinoma formation (35–37). Similarly, conditional deletion of *Ctnnb1* exon 3 in mouse hepatocytes can induce the formation of HCC or hepatoblastoma-like tumors (38).

The therapeutic potential of targeting the WNT/β-catenin pathway was assessed by reversible loss-of-function genetic approaches. The reversibility of shRNA-mediated Apc knockdown allowed Dow and colleagues (35) to demonstrate that Apc loss is required for tumor maintenance in the intestine. When Apc expression was restored in early-stage or even more aggressive adenomas carrying *Kras*^G12D^ mutations and *p53* loss, rapid and complete tumor regressions were observed. Importantly, tumor recurrence was not observed over prolonged observation periods of mice in which Apc expression had been restored (35). To model the metastatic disease, Dow and colleagues switched to orthotopic transplantation of organoids derived from colorectal cancer GEMMs carrying inducible *Apc* shRNA, *Kras*^G12D^ mutations, and *p53* loss (39). Apc restoration, even in the metastatic setting, modeled through spleen injection of the organoids, was sufficient to suppress the WNT/β-catenin pathway and induce cellular differentiation (39), suggesting that these models will be suitable to help drive the development of therapeutics for the treatment of metastatic colorectal cancer.

The therapeutic potential of targeting β-catenin was also demonstrated in HCC using lipid nanoparticle (LNP)–encapsulated siRNA targeting *CTNNB1* (LNP-CTNNB1) across various humanized mouse models representing *CTNNB1*-mutant HCC, in which degradation-resistant CTNNB^T41A^ or CTNNB1^S45Y^ mutants were coexpressed either with the *NFE2L2*^*G13A*^ mutant, coding for a stabilized variant of the nuclear factor erythroid 2–related factor 2 (NRF2) transcription factor, or with the tyrosine kinase MET (40). Treatment of mice with early-stage tumors with LNP-CTNNB1 resulted in reduced tumor growth; however, it restarted as soon as treatment was stopped. Treatment of mice with tumors at later stages was still efficacious, although to a lesser extent, possibly due to insufficient knockdown of *CTNNB1* in larger tumors. Interestingly, the response to RNAi-mediated knockdown of *CTNNB1* was associated with boosted adaptive immunity, and combination with an immune checkpoint inhibitor (anti–PD-1 inhibitor) further improved the response, consistent with previous work demonstrating that β-catenin activation in HCC mouse models promoted immune evasion and resistance to anti–PD-1 (19, 40).

Collectively, there is ample evidence from GEMMs and other mouse models supporting the notion that targeting β-catenin and the WNT pathway is a valuable therapeutic opportunity for metastatic colorectal cancer and HCC and potentially for WNT-driven cancers irrespective of tissue origin.

## Challenges in Drugging β-Catenin–Driven Cancers

Despite intense efforts over the past two decades, attempts to drug the WNT/β-catenin pathway have largely been unsuccessful. WNT ligand– and receptor–targeting agents that intercept the pathway upstream of relevant mutations have displayed minimal efficacy and narrow therapeutic windows based on clinical trials reported so far. Some of the most common serious adverse events are related to bone and gastrointestinal (GI) toxicity, including loss of bone density, bone fractures, dysgeusia, and diarrhea (41–43). These are all considered on-target/off-tissue toxicities, consistent with the crucial role of the pathway in bone and intestinal stem cells (44).

Recent reviews by Parsons and colleagues (3) and by Wu and colleagues (45) have comprehensively summarized clinical trials of these agents, including the PORCN inhibitor WNT974, vantictumab targeting multiple FZD receptors, BI 905677 targeting LRP5/6, as well as OMP-131R10, which targets RSPO3; see Fig. 1. Other approaches in clinical studies may lack target and pathway specificity, such as the CBP inhibitor E7386, which has potentially limited specificity for targeting the CBP/β-catenin transcription complex (46).

## Recent Progress in Directly Targeting β-Catenin

A range of new therapeutic concepts directly targeting β-catenin are now underway and are summarized in Fig. 1 and Table 1. The most advanced approaches are highlighted and include cell-penetrating peptides, condensate modulators (c-mod), and siRNAs targeting β-catenin. Small-molecule inhibitors/degraders are less advanced. The difficulty in identifying functional direct small molecule β-catenin inhibitors has recently been highlighted by Kessler and colleagues (61). One avenue to overcome this druggability challenge is to intervene by generating larger molecules, for example, cell-penetrating peptides, first pioneered by Walensky (62) and explored by Grossmann in the Verdine group (62). The initial discovery of a hydrocarbon-stapled peptide that directly targets β-catenin and interferes with its ability to serve as a transcriptional coactivator for TCF proteins (63) has laid the foundation for optimization efforts of such peptides for clinical testing. FOG-001 (INN: zolucatetide) is a novel, synthetic, conformationally constrained α-helical peptide designed to bind β-catenin. FOG-001 potently and selectively disrupts the interaction of β-catenin with its obligate binding partner, the transcription factor TCF4 (encoded by the *TCF7L2* gene). Preclinical studies in patient-derived xenograft (PDX) models of colorectal cancer and other solid tumors harboring WNT/β-catenin pathway activating mutations have shown that treatment with FOG-001 alone leads to potent tumor growth inhibition and regression. Combinatorial efficacy was observed in preclinical models when FOG-001 was coadministered with chemotherapy (5-fluorouracil) or inhibitors of VEGF or PD-1 (47, 48).

**Table 1.** Current status of Wnt/β-catenin targeting agents.

Compound	Company	Target (drug class)	Mechanism of action	Development phase; route of administration	Cancer type	CT number(s); accessed June 17, 2026	Reference(s)
FOG-001	Parabilis Medicines	β-catenin/TCF4 (peptide)	Peptide blocking the β-catenin–TCF4 interaction	Phase I/II; i.v. admin	Solid tumors with WNT pathway activating mutations	NCT05919264	(47–50)
ST316	Sapience Therapeutics	β-catenin/BCL9 (peptide)	Peptide blocking the β-catenin–BCL9 interaction	Phase I/II; i.v. admin	Solid tumors with abnormalities of the WNT/β-catenin signaling pathway	NCT05848739	(51, 52)
DPTX3186	Dewpoint Therapeutics	β-catenin (small molecule)	β-catenin modulation via biomolecular condensates	Phase I; oral admin	Wnt-pathway activated solid tumors	NCT07312903	(53–55)
ALN-BCAT	Alnylam Pharmaceuticals	β-catenin (siRNA)	Reduction of *CTNNB1* mRNA	Phase I; i.v. admin	HCC	NCT06600321	(56)
E7386	Eisai/Prism	β-catenin/CBP (small molecule)	Small molecule unspecifically blocking the β-catenin/CBP interaction	Phase I/II; oral admin	Endometrial cancers, solid cancers	NCT04008797, NCT05091346 and others	(46)
β-catenin degrader	Parabilis Medicines	β-catenin/TCF4 (PROTAC peptide)	Bifunctional peptidic β-catenin degrader	Preclinical	​	​	(57, 58)
EN83	Nomura Lab	β-catenin (small molecule)	Covalent degrader of β-catenin engaging unknown E3 ligase	Preclinical	​	​	(59)
CTX-B-330	Coltac Therapeutics	β-catenin (small molecule)	Molecular glue enhancing binding of β-catenin to its E3 ligase β-TrCP	Preclinical	​	​	(60)
WNT974	Novartis	PORCN (small molecule)	Suppression of WNT secretion by preventing O-linked palmitoleoylation of WNT ligands	Discontinued	​	​	(3, 45)
Vantictumab	OncoMed Pharmaceuticals	Multiple FZD proteins (mAb)	Antibody blocking FZD 1, 2, 5, 7, 8	Discontinued	​	​	(3, 45)
BI 905677	Boehringer Ingelheim	LRP5/6 (mAb)	Antibody blocking LRP5 and LRP6	Discontinued	​	​	(3, 45)
OMP-131R10	OncoMed Pharmaceuticals	RSPO3	Antibody blocking RSPO3	Discontinued	​	​	(3, 45)

Preliminary safety and efficacy data from a phase I/II trial of FOG-001 in patients with solid tumors harboring WNT/β-catenin pathway activating mutations were presented at the ESMO and AACR-NCI-EORTC 2025 meetings (49, 50). Based on a study of 83 patients, FOG-001 monotherapy was well tolerated, with a manageable safety profile for all doses tested. Unlike previous WNT pathway inhibitors (e.g., PORCN, FZD), no high-grade bone toxicity has been observed. No high-grade GI toxicities and no high-grade dysgeusia were observed. On-target pharmacodynamic effects, as well as initial antitumor activity, were reported, showing that FOG-001 is a *bona fide* β-catenin inhibitor.

Importantly, preliminary antitumor activity of FOG-001 was observed in desmoid tumors, a rare, locally invasive soft-tissue tumor type with a substantial impact on the quality of life of affected patients. As mentioned above, desmoid tumors almost universally express activated β-catenin due to stabilizing hotspot mutations in exon 3 (T41A/S45F; ref. 9). Among patients with desmoid tumors, all response-evaluable patients *n* = 10) experienced tumor reduction. In particular, an 80% objective response rate (ORR) was observed in patients with more than one postbaseline scan (*n* = 5). FOG-001 additionally yielded single-agent activity in other low mutational complexity tumor types in which WNT/β-catenin mutations are thought to be the primary drivers of disease, including adamantinomatous craniopharyngioma (ACP), salivary gland cancer, and solid pseudopapillary neoplasm. In addition, a durable response to FOG-001 was described in a patient with recurrent ameloblastoma characterized by a pathogenic *APC* frameshift mutation (64). Furthermore, the first clinical evidence was presented that FOG-001 also reduced polyp burden in a patient with familial adenomatous polyposis, a rare disorder caused by germline loss-of-function mutations in the *APC* gene (https://parabilismed.com/press-release/parabilis-medicines-reports-early-clinical-evidence-that-zolucatetide-reduces-polyp-burden-in-a-patient-with-familial-adenomatous-polyposis/). In a cohort of 22 patients with efficacy-evaluable microsatellite stable (MSS) colorectal cancer treated at an efficacious dose range, a 50% disease control rate was observed (50). Paired biopsy studies suggested an exposure-related inhibition of WNT/β-catenin signaling and provided a dataset informing opportunities for combination development. Interestingly, the level of WNT pathway inhibition (as determined by a WNT gene signature score) correlated with increasing inhibition of stemness-related genes and induction of differentiation programs in these treated tumors. Patients with MSS colorectal cancer are treated with chemotherapy as the standard of care, with superior responses typically observed in tumors exhibiting more differentiated, less stem cell–like transcriptional programs. Similarly, the induction of genes involved in angiogenesis that was observed in this analysis is a known feature of WNT/β-catenin inhibition and may prime these tumors for enhanced sensitivity to antiangiogenic combination partners. Moreover, the induction of a tumor inflammatory signature—including upregulation of genes involved in antigen presentation and T-cell recruitment within the tumor microenvironment—suggests therapeutic combinability with immune checkpoint inhibitors, which have thus far demonstrated limited activity as monotherapies in MSS colorectal cancer (49). Collectively, these initial clinical data seem clinically meaningful and support the ongoing expansion of FOG-001 in desmoid tumors, whereas the accompanying translational studies provide a rationale for mechanism-based combination strategies in MSS colorectal cancer.

ST316 is a second novel, cell-penetrating peptide antagonist of β-catenin that is currently being tested in clinical trials. Instead of addressing the β-catenin–TCF4 interaction, ST316 blocks the interaction of β-catenin with B-cell lymphoma 9 (BCL9), a transcriptional cofactor that drives distinct but partially overlapping transcriptional programs compared with the TCF proteins (65). An initial safety and biomarker assessment was recently reported based on a phase I/II dose escalation–expansion study encompassing 23 patients with solid tumors, including colorectal cancer. Most adverse events were described as mild to moderate, and no dose-limiting toxicities were observed. No high-grade bone, GI, or taste bud toxicities were described. Single-agent ST316 yielded disease stabilization in a small number of patients, suggesting early hints of activity. Paired biopsy data suggested that ST316 maintains β-catenin in the cytoplasm and prevents its entry into the nucleus. In addition, changes in immunosuppressive myeloid cells were reported, consistent with mechanistic evidence of β-catenin antagonism (51). Phase II clinical data for the β-catenin antagonist ST316 showed a confirmed 47% ORR and a 93% disease control rate when combined with fluorouracil, leucovorin, and irinotecan chemotherapy + bevacizumab in second-line metastatic colorectal cancer (52). Although this initial ORR compares favorably with historical benchmarks for anti–VEGF-chemotherapy combinations in second line (2L) colorectal cancer, further biomarker and efficacy data in a randomized study are awaited to establish the clinical benefit of ST316.

DPTX3186 is a small-molecule c-mod that acts via a novel mechanism of action, sequestering β-catenin into inactive condensate depots (53). Biomolecular condensates are transient liquid-like droplets that can organize proteins within the cell. Based on a condensate phenotypic screen, compounds were identified that can sequester β-catenin in transcriptionally inactive nuclear condensate depots. The development candidate DPTX3186 has been shown to induce β-catenin depot formation, downregulate β-catenin target gene expression, and reduce the viability of colorectal cancer cell lines. Notably, DPTX3186 exhibited cancer cell killing across a gastric cancer cell panel with diverse molecular profiles. In a panel of gastric cancer PDX models, DPTX3186 treatment resulted in antitumor activity and decreased β-catenin–regulated target expression (53). Apparently, DPTX3186 does not directly bind β-catenin, and its target has not been disclosed up to now (54). In a recent report, DPTX3186 was profiled across a diverse panel of 20 gastric cancer PDX models. A six-gene mutation criterion (*ARID1A*, *PIK3CA*, *SPEN*, *CTNNB1*, *APC*, and *RNF43*) enriched for higher antitumor efficacy and comprised a predicted gastric cancer prevalence of more than 40% (55). A phase I clinical trial of DPTX3186 has recently been announced in subjects with WNT-pathway activated solid tumors for which no other treatments exist (NCT07312903).

ALN-BCAT comprises a chemically modified siRNA encapsulated in a LNP formulation that selectively reduces *CTNNB1* mRNA and thereby β-catenin protein levels, as well as downstream biomarkers and tumor cell proliferation in *in vitro* model systems. Antitumor activity has been observed in multiple HCC mouse models, associated with changes in β-catenin–related biomarkers (56). Moreover, ALN-BCAT, as well as a related RNA interference therapeutic strategy termed LNP-CTNNB1, both induced changes in the tumor microenvironment in HCC organoids and mouse immunocompetent HCC models, with increases in CD4^+^ and CD8^+^ T cells and macrophages repolarizing toward an M1-like phenotype, consistent with a shift to a more immune-stimulatory antitumor microenvironment (56). ALN-BCAT is currently being evaluated in a phase I study in patients with advanced or metastatic HCC, both as monotherapy and in combination with the PD-1 inhibitor pembrolizumab. RNA interference has been shown to act as an effective therapeutic modality in liver disease, with several therapies already approved and many more in development or clinical trials (66). The liver is an ideal target for RNAi due to the development of specific delivery systems that target hepatocytes. Although this therapeutic modality has a strong rationale in early-stage HCC harboring *CTNNB1* activating mutations, it remains to be shown whether this approach will prove effective in patients with disseminated metastatic disease. Based on data shown by Lehrich and colleagues (40), RNAi-mediated inhibition of β-catenin may have the potential to provide antitumor effects as a monotherapy in early-stage disease or in a neoadjuvant setting in patients with WNT-β-catenin–active liver tumors. These studies also support the clinical investigation of RNAi therapeutic approaches targeting β-catenin in combination with immune checkpoint inhibitors in patients with advanced-stage WNT/β-catenin active HCC.

The early clinical data around these novel agents directly targeting β-catenin suggest better tolerability compared with past approaches. Nevertheless, it is important to consider that systemic inhibition of β-catenin still bears the risk of causing adverse events related to the crucial role of the WNT pathway in adult stem cells. These toxicities are likely to be similar to those caused by agents targeting WNT ligands or receptors, mentioned above. Careful evaluation of emerging safety data will be necessary to guide the implementation of risk-mitigation strategies, including optimized dosing regimens, tumor-selective delivery approaches, and other interventions designed to broaden the therapeutic window.

## Toward Restoring Cellular Protein Homeostasis of β-Catenin

Reestablishing cellular degradation of β-catenin is an attractive therapeutic concept, as degraders may be particularly efficacious in cancers harboring genetic aberrations in the β-catenin degradation machinery. A first-generation peptidic proteolysis-targeting chimera (PROTAC)-like β-catenin degrader, xStAx-VHLL, has been described as achieving efficient target degradation and restraining cancer cell proliferation (57). Building on this early peptidic PROTAC degrader, Parabilis is developing hetero-bifunctional degraders, consisting of a β-catenin–binding helicon-type peptide linked to a small-molecule ligand of an E3 ligase (https://parabilismed.com/innovation/programs; refs. 54, 58). The Nomura lab has reported the monovalent degrader EN83 that acts through the targeting of cysteine 619 on β-catenin and leads to its depletion in a ubiquitin-proteasome–dependent manner (59). Recently, a small molecule termed CTX-B-330 was identified in a screen for molecular glues that promote proteasome-dependent degradation of β-catenin. Interestingly, CTX-B-330 drives ternary complex formation between β-catenin and its E3 ligase β-TrCP, resulting in targeted degradation of β-catenin with minimal impact on other β-TrCP substrates. CTX-B-330 showed preferential antiproliferative activity in WNT/β-catenin pathway mutated cell lines, patient-derived organoids, as well as in a colorectal cancer xenograft model (60). Further efforts will be required to advance peptide-based or small molecule–based β-catenin degraders into clinical development.

Targeting mutationally stabilized β-catenin for degradation is an attractive therapeutic strategy, with the potential for a favorable therapeutic window given the tightly regulated, low basal levels of the protein in normal tissues. Degraders may, however, carry additional significant toxicity risks due to the unintended degradation of the membrane as well as the nuclear pool of β-catenin. Membrane-associated β-catenin plays a critical structural role in adherens junctions (67). Physiologically, the most relevant phenotype linked to the loss of membrane β-catenin is the disruption of the blood–brain barrier (BBB). In adult mice, endothelial-specific deletion of the *Ctnnb1* gene resulted in BBB leakage and severe neurologic symptoms, including seizures, brain hemorrhages, inflammation, and ultimately postictal death within 16 days (68). During compound optimization, the activity and potency of degraders will need to be carefully tuned to avoid degradation of the E-cadherin (membrane)–bound pool of β-catenin, at least in endothelial cells.

## Conclusions and Future Directions

Effectively addressing β-catenin still poses significant challenges due to its “undruggable” nature, attributed to the lack of apparent drug-binding pockets and the highly extended binding interface with the TCF family of transcription factors (63). However, emerging research has yielded various novel strategies to target β-catenin, including direct engagement with cell-penetrating α-helical peptides, molecular c-mods, as well as RNA interference–based approaches to reduce β-catenin activity. Several of these agents have now entered the clinic. An important advance is the early clinical efficacy observed with zolucatetide (also known as FOG-001) in patients with WNT/β-catenin pathway–activated cancers. These data demonstrate the potential to directly and effectively address this elusive cancer driver, at least in patients with low mutational burden characterized by prevalent β-catenin stabilizing mutations, including desmoid and ACPs. Current research continues to explore further novel strategies to target WNT/β-catenin–driven cancers. PROTACs and other types of degraders offer innovative approaches to restore β-catenin degradation, which is abolished in WNT/β-catenin–driven cancers, but their preclinical development requires further studies to demonstrate efficacy and ensure safety. The therapeutic efficacy of β-catenin–targeting drug candidates in complex cancers harboring WNT/β-catenin pathway mutations, which are characterized by high mutation rates, multiple additional cancer drivers and a high level of tumor heterogeneity, has yet to be fully established in a clinical setting. These mutationally complex cancer types, with frequent pathway mutations yet multiple overlapping cancer dependencies, include colorectal cancer, HCCs, prostate cancer, non–small cell lung cancer (NSCLC), melanoma, and endometrial cancer, as outlined above. Initial data reported for FOG-001 in a cohort of patients with colorectal cancer suggest disease stabilization, and accompanying biomarker analyses have provided mechanistic insights supporting multiple rational combination strategies, including chemotherapy, anti-VEGF therapy, and anti–PD-1 therapy (49). Preclinical data also support a combination approach with emerging KRAS inhibitors in colorectal cancer (69). It remains to be determined if more potent β-catenin drug candidates will be able to induce tumor regressions as monotherapy in this setting. Based on the high prevalence (dominated by *APC*, ∼69%) of WNT pathway mutations (∼120,000 newly diagnosed patients/year in the United States), colorectal cancer will be of central interest in further studying the clinical utility of β-catenin–targeting approaches. Further studies are required to determine patient stratification markers beyond WNT/β-catenin pathway alterations. These will need to consider the presence of specific comutations (e.g., *KRAS/BRAF/TP53/SMAD4* mutational status), as well as subtype classification based on histology and/or molecular classification, for example, the consensus molecular subtype 2 (CMS2), which is characterized by marked WNT signaling activation (70).

WNT/β-catenin activation is observed in close to 39% (dominated by *CTNNB1*, ∼31%) of all patients with HCC, with 13,000 patients/year in the United States. In this cancer type, established first-line therapies are centered around combinations of immune checkpoint inhibitors. Notably, FOG-001 has been profiled across a range of HCC PDX models, collectively indicating profound antitumor efficacy (48). WNT/β-catenin–active HCCs are of particular interest based on the prevalence of β-catenin oncogenic stabilizing mutations but also based on extensive data suggesting a key role of β-catenin in immune cell exclusion (40, 71).

Based on mutational analyses, patient populations addressable by β-catenin–targeted drugs include NSCLC (combined WNT pathway gene prevalence 6%; ∼10,000 patients/year in the United States), castration-resistant prostate cancer (CRPC, ∼3,300 to 6,600 patients with CRPC/year in the United States with WNT pathway alterations; assuming combined WNT pathway gene prevalence of ∼10% in prostate—actual prevalence most likely higher in CRPC settings—and 10% to 20% progression from incident prostate cancer shown in Fig. 2A), endometrial cancer (combined WNT gene prevalence 23%; ∼16,000 patients/year in the United States), as well as a range of additional solid cancers. In principle, tumors driven by ligand-dependent activation of the WNT pathway are also expected to respond to β-catenin–targeted therapies, thereby representing an opportunity to broaden the clinical impact of these agents. Additional studies are required to better understand how β-catenin signaling varies across tissues and cell types and how this influences the effect of *APC/CTNNB1* pathway mutations on the cancers derived from these tissues. The response to inhibitors of the WNT pathway will likely also be modulated by the presence or absence of comutations, including actionable driver mutations, such as RTK/KRAS mutations in NSCLC and androgen receptor and PTEN loss in CRPC, as well as the mutation status of *TP53* and other tumor suppressor genes.

Broad profiling of drug candidates across cell panels, organoids, and xenograft models representing diverse cancer subtypes could help in identifying the most promising opportunities for monotherapy clinical development. Combination studies with other molecularly targeted therapies (based on cancer codependencies), immune checkpoint inhibitor therapies, and emerging antibody–drug conjugates, bispecific antibodies, and other innovative treatment modalities, including T-cell receptor–engineered T cells targeting mutant β-catenin antigens bound to HLA molecules (72), will further fuel precision combination strategies. Genomic profiling and molecular diagnostics are crucial for identifying pathway mutations, understanding determinants of molecular response and resistance, and guiding therapeutic choices.

Targeting β-catenin has been a long-standing objective in cancer research. Over the past several years, intensified efforts have yielded tangible progress and initial breakthroughs. Nevertheless, further advances enabling more potent and durable targeting of β-catenin will be required to expand precision therapy options to the broadest possible population of patients with WNT/β-catenin–driven cancers.
